# Restoring p53 Function in Head and Neck Squamous Cell Carcinoma to Improve Treatments

**DOI:** 10.3389/fonc.2021.799993

**Published:** 2022-01-06

**Authors:** Tycho de Bakker, Fabrice Journe, Géraldine Descamps, Sven Saussez, Tatiana Dragan, Ghanem Ghanem, Mohammad Krayem, Dirk Van Gestel

**Affiliations:** ^1^ Department of Radiation Oncology, Institut Jules Bordet, Université Libre de Bruxelles, Brussels, Belgium; ^2^ Laboratory of Clinical and Experimental Oncology (LOCE), Institut Jules Bordet, Université Libre de Bruxelles, Brussels, Belgium; ^3^ Laboratory of Human Anatomy and Experimental Oncology, Faculty of Medicine and Pharmacy, University of Mons, Mons, Belgium

**Keywords:** p53, mutation, HPV, HNSCC, targeted therapy

## Abstract

TP53 mutation is one of the most frequent genetic alterations in head and neck squamous cell carcinoma (HNSCC) and results in an accumulation of p53 protein in tumor cells. This makes p53 an attractive target to improve HNSCC therapy by restoring the tumor suppressor activity of this protein. Therapeutic strategies targeting p53 in HNSCC can be divided into three categories related to three subtypes encompassing WT p53, mutated p53 and HPV-positive HNSCC. First, compounds targeting degradation or direct inhibition of WT p53, such as PM2, RITA, nutlin-3 and CH1iB, achieve p53 reactivation by affecting p53 inhibitors such as MDM2 and MDMX/4 or by preventing the breakdown of p53 by inhibiting the proteasomal complex. Second, compounds that directly affect mutated p53 by binding it and restoring the WT conformation and transcriptional activity (PRIMA-1, APR-246, COTI-2, CP-31398). Third, treatments that specifically affect HPV^+^ cancer cells by targeting the viral enzymes E6/E7 which are responsible for the breakdown of p53 such as Ad-E6/E7-As and bortezomib. In this review, we describe and discuss p53 regulation and its targeting in combination with existing therapies for HNSCC through a new classification of such cancers based on p53 mutation status and HPV infection.

## Introduction

Head and neck squamous cell carcinoma (HNSCC) is diagnosed in 890.000 patients each year worldwide, ranking it as the sixth most common cancer in the world (1). HNSCC is a collection of cancers encompassing the oral cavity, nasopharynx, oropharynx, hypopharynx and larynx (2). The most common genetic aberrations are the overexpression of the epidermal growth factor receptor (EGFR) and inhibiting mutations of p53 in 95% and 75-85% of non-HPV-related cases respectively (3). Additionally, the PI3K/AKT/mTOR signalling pathway is the most commonly mutated signalling pathway with up to 62% of HNSCC patients showing activating mutations. The hyperactivation of this pathway contributes to increased cell growth, proliferation and cellsurvival as well as regulation of apoptosis and DNA damage repair (4).

**Figure 1:**
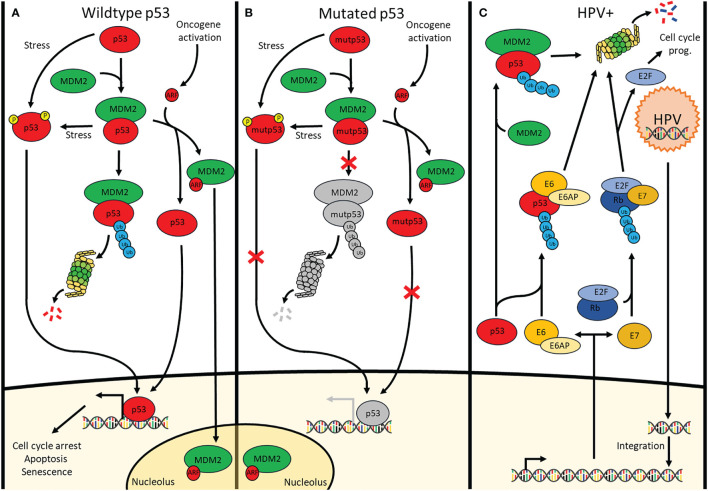
Signalling pathways of p53 in cells containing **(A)** wildtype p53 cells, **(B)** mutated p53 cells and **(C)** HPV-positive cells. **(A)** p53 is continuously polyubiquitinylated by MDM2 under physiological conditions. Additionally, there is MDM4 (Not shown) which inhibits p53 through direct interaction. Stressors cause p53 phosphorylation preventing or removing the binding of MDM2 & MDM4 to p53 allowing downstream transcription activation. Upon oncogene activation, the INK4A locus is transcribed, one of its products being ARF which sequesters MDM2 in the nucleolus thus releasing p53 allowing it to transcribe its target genes involved in cell cycle arrest, apoptosis and senescence. **(B)** Although mutated p53 undergoes the same pathways as wildtype p53, the mutation causes a lack of downstream transcription activation. **(C)** Upon infection by HPV, the viral DNA integrates into the genome where it is transcribed for the production of new viral particles. Viral E6 and E7 enzymes are transcribed as well causing the breakdown of p53 and Rb, respectively. This respectively results in loss of apoptosis induction and uncontrolled cell cycle progression.

Due to the prevalence of p53 mutation, p53 is a frequent topic of many HNSCC studies. A large portion of those reports involves the reactivation of p53 which has become a promising treatment approach in combination with targeted therapies for HNSCC (5). In addition, p53 is responsible for the transcription activation of many genes involved in cell cycle arrest, apoptosis, senescence, DNA repair and metabolism (6). Indeed, activation of p53 (**Figure 1A**) occurs when either the cell accumulates too much DNA damage, an oncogene is activated, or in case of stressors such as nutrient deprivation or hypoxia (7, 8). Depending on the severity of these activators, the cell will undergo different responses such as induction of cell cycle arrest, apoptosis, senescence, induction of protective antioxidant activities, DNA repair as well as the alteration of the mitochondrial respiration (9). In cancer cells without functional p53, the cell has a deletion in one of the *TP53* alleles and a mutated, non-functional form of *TP53* in the other allele. Consequently, the cell can accumulate additional mutations, without resulting in apoptosis or cell cycle arrest for repair, and further activate oncogenes that result in oncogenesis (10) (**Figure 1B**).

In cells with wild-type p53 (WT p53), double- and single-stranded breaks in the DNA activate kinases such as ataxia-telangiectasia mutated (ATM) and ATM and Rad3 related (ATR), respectively (11), which in turn directly phosphorylate p53 at Ser-15 and will activate checkpoint kinase 2 (CHK2) and checkpoint kinase 1 (CHK1), respectively. The latter will subsequently phosphorylate Ser-20 of p53, inducing a change in its conformation and therefore removing inhibition of p53 by mouse double minute 2 (MDM2) which dissociates from p53 (12, 13). Additionally, ATM phosphorylates MDM2 and MDM4/X lead to the degradation of both of these p53 inhibitors, once again activating p53 (14). Under normal conditions, MDM2 continually causes polyubiquitination resulting in the proteasomal breakdown of p53. By contrast, MDM4/X binds p53 to inhibit its function as a transcription factor albeit without inducing the degradation of p53 (15). Upon activation of an oncogene, the Cyclin-Dependent Kinase Inhibitor 2A (*CDKN2A*) locus gets activated. This occurs through the inhibition of the histone modifier Enhancer of zeste homolog 2 (EZH2) causing derepression of the *CDKN2A* locus. Activation of this locus results in the expression of both p16, also known as INK4A, as well as p14, also known as ADP-ribosylation factor(ARF). ARF, in turn, binds MDM2 causing its dissociation from p53 and translocation and sequestration into the nucleolus, thereby activating p53. At the same time, p16 binds and inhibits Cyclin-dependent kinase 4/6 (CDK4/6) which prevents retinoblastoma protein (Rb) phosphorylation and thus also prevents the release of E2 factor (E2F), preventing cell cycle progression (16). One of p53’s target genes is p21, a protein with a similar function to p16, also causing inhibition of E2F and preventing cell cycle progression (17). The combination of p16 & p53 activation causes irreversible cell cycle arrest also known as senescence (18). In about 50% of cancers, p53 is mutated and in most of the other cancers, other parts of the p53 pathway are defective resulting in reduced or no p53 signalling (19, 20). This occurs through mechanisms such as overexpression of MDM2 or MDM4/X, downregulation of ARF or deregulation of microRNAs (21). In HNSCC, there are other ways in which p53 is inactivated besides inactivating mutations of p53. Based on data acquired from a 243-patientcohort collected by the cancer genome atlas (22) the highest percentage of mutations causing p53 inactivation in human papilloma virus (HPV) negative HNSCC occurs in *TP53* itself (84%) followed by *CDKN2A* (57%) which either contains a homozygous deletion or a mutation (22). A recent computational analysis of the TCGA database revealed that, although more or less severe depending on the types of mutations and localization of the mutation within the gene, patients with mutated *TP53* HNSCC were shown to have reduced overall survival (23). The 8 most common p53 mutations are all located in the DNA binding region which can be found between residues 103-292 (3) Among these mutants are 2 groups, a group that has mutated the specific residues responsible for binding the DNA and a second group that changes the conformation of the DNA binding element thereby also preventing DNA binding (24). Some of the treatments in this review specifically target these mutated conformations to induce the refolding into their original wild-type conformation. Other treatments target cofactors associated with p53 in order to result in reactivation of p53 (**Table 1**). Evidently, the mutational status of p53 in cancer that is to be treated is important for the choice of treatment that is to be used. While many different mechanisms for treatments have been targeted, not all treatment modalities have the same efficacy. The 84% mutation rate of p53 as well as the worse prognosis in HNSCC connected to these mutations warrant research towards a treatment capable of reactivating p53.

**Table 1:** The different types of molecules and mechanisms used to reactivate p53 by targeting either p53 directly or another protein preventing p53 activity.

Drug	Molecule type	Target	HNSCC subtype	P53 independent effects
Ad-E6/E7-AS	Viral particle	E6/E7 RNA transcript	HPV-positive	/
Ad-p53	Viral particle	P53	mutated p53	/
Bortezomib	Small molecule	26S proteasome	HPV-positive	NFkB inhibition
CH1iB	Helical mimic	P300	WT p53	/
COTI-2	Small molecule	P53	Mutated p53	Glutathione depletion PI3Ka signalling inhibition
CP-31398	Small molecule	P53	Mutated p53	ROS generation
Nutlin-3	Small molecule	MDM2	WT p53	/
ONYX-015	Viral particle	^mut^ p53 cells	Mutated p53	/
PM2	Stapled peptide	MDM2	WT p53	/
PRIMA-1 & APR-246	Small molecule	P53	Mutated p53	ROS generation
RITA	Small molecule	P53	Mutated p53	JNK activation
Triptolide	Small molecule	E6 transcription	HPV-positive	HSP7 inhibition

Treatments are divided based on the HNSCC subtype which they are most suited to treat. Some treatments have additional alternative effects that do not involve p53.

HPV is the cause of a portion of HNSCC, particularly in the oropharynx. In recent years, HPV infection has become more and more prevalent (25) and might soon take the place of the most common cause of HNSCC from chronic exposure to tobacco and alcohol. Additionally, the average age of HPV-related head and neck cancer is significantly lower than that of non-HPV-related HNSCC (26). However, HPV-related cases of HNSCC have a more favorable prognosis than non-HPV-related HNSCC (27). Besides the difference in prognosis, the fundamental mechanism causing HNSCC is also different. The human papillomavirus contains 6 early proteins and 2 late ones. These late proteins are responsible for the capsid synthesis which is needed for the envelopment of the virus upon its duplication. The early proteins are involved in the initial steps of the infection, and include E1, E2 and E4 which are responsible for viral genome replication, viral DNA replication and transcription, and packing of the viral genome by late protein L1 and L2. However, some early proteins are known to be oncoproteins. Cellular transformation is mainly caused by the two viral proteins E6 and E7 (**Figure 1C**). In this context, E6 interacts with E6 associated protein (E6AP) and functions as a ubiquitin ligase specifically for p53 degradation. Nevertheless, the p53-E6AP-E6 complex does not always lead to degradation as p53 has 2 domains with which it interacts within the complex. There is a high-risk HPV-E6 which binds both a C-terminal binding domain not leading to degradation as well as a core-binding domain which does lead to degradation. By contrast, the low-risk HPV-E6 only binds the C-terminal domain and thus does not induce the degradation of p53. With high-risk HPV, E6 can lead to accumulation of mutations, eventually leading to the activation of oncogenes and the transformation into cancer cells (28). Simultaneously, E7 causes the breakdown of Rb which leads to a lack of cell cycle arrest and thus continued cell cycle progression once again contributing to oncogenesis. Of note, E7 contributes to accumulating p16 in the cytoplasm and nucleus of cancer cells, such accumulation being viewed as a master predictive marker of a transcriptionally active HPV infection.

Since HNSCC encompasses tumors in many different sites, treatment may be slightly different depending on the site. In general, the treatment schemes by stage are as follows. Treatment for early-stage disease (stage I-II) consists of either surgery or RT (29). Depending on adverse features in the resection, adjuvant radiotherapy +/- chemotherapy is advised (29). Advanced disease (stage III-IVa/b) is treated with the combination of surgery and postoperative RT or by post-operative chemoradiotherapy (CRT) in case of high risk factors (extracapsular extension and/or R1 resection) (29). Concomitant chemoradiotherapy (CCRT) is indicated when the patient is inoperable, in case of organ preservation or when surgery is considered to be too mutilating. Stage IVc cancers are highly influenced by the performance score and comorbidities which can call for palliative local or systemic treatment (30). The advent of T-cell-based immunotherapies in recent years now allows us to improve overall survival of recurrent HNSCC in approximately 20% of these patients (31, 32). Pembrolizumab and Nivolumab are immune checkpoint inhibitors whose mode of action is to reactivate cytotoxic T cells so that they can kill tumor cells. However, resistance to these treatments which is related to the immunosuppressive tumor microenvironment (TME) led to clinical trials combining immunotherapeutic treatment with targeting of the TME (33). Over the past decade, in young patients affected by an HPV+ oropharyngeal carcinoma, a treatment de-escalation program is under research to avoid the harmful side effects of cisplatin and radiotherapy in this population. This concept mainly consists of reduced radiotherapy doses, using less toxic chemotherapy agents such as Cetuximab or even removing chemotherapy administration (34). So far, this approach has been disappointing.

In this review, we will summarize the p53 reactivating treatments reported to get an anti-HNSCC activity *in vitro*, *in vivo* and/or in clinical settings. In addition, based on p53 mutational status and HPV infection, we will propose a new classification for HNSCC encompassing 1°) wildtype p53, 2°) mutated p53, and 3°) HPV-positive head and neck carcinomas, related to specific treatment strategies targeting p53 in order to restore its activity.

## Molecular Compounds that Primarily Target Mutated p53 HNSCC

From large screening analyses, several small molecules were identified to directly interact with p53 and restore its activity. In many cases, such compounds had also p53-independent effects that may contribute to the antitumor effect in many cancers including HNSCC.

### COTI-2

In 2016, a compound screening known as CHEMSAS discovered a new compound, COTI-2 (35), which is a third-generation thiosemicarbazone and has both a p53 dependent as well as a p53 independent mechanism to affect cancer cells. Although the exact mechanism for both modes of action is not well known, the independent mechanism involves downregulation of the PI3K/AKT/mTOR signalling pathway (5, 36). When comparing the IC50 of cell lines containing WT p53 versus mutated p53, it was observed that cell lines with mutated p53 were more sensitive to COTI-2 than cells with WT p53. Additionally, by using conformation-dependent antibodies, it was determined that COTI-2 could cause refolding of unfolded or misfolded p53 into a wild-type conformation (37). Using surface plasmon resonance, Synnot et al. evaluated the binding affinity of immobilized p53 with different concentrations of COTI-2. There it was seen that COTI-2 interacts with both the full-length p53 in addition to the DNA binding domain of p53 (37). The inherent properties of a thiosemicarbazone is its ability to form complexes with copper ions. These copper complexes subsequently interact with glutathione (GSH) which then allows the complex to be exported by the ABCC1, conferring resistance of the cell to COTI-2 (38).

COTI-2 shows both synergistic effects with cisplatin and radiotherapy in the treatment of HNSCC in both *in vitro* and *in vivo* setting (5). Currently, COTI-2 is being evaluated in a clinical trial encompassing gynaecological cancers, colorectal cancer, lung cancer and head and neck squamous cell carcinoma (NCT02433626) (39). However, at the time of writing, no results have been posted yet.

### CP-31398

In 1999, a compound screening for protection of p53 from thermal denaturation was performed for a collection of 100.000 small molecules (40). Using two antibodies, mAb240 which binds an epitope only accessible when p53 is in an inactive conformation and mAb1620 which binds an epitope that becomes accessible upon p53 taking on the active conformation (34),Foster et al (40) showed that addition of CP-31398 (*N*′-[2-[2-(4-methoxyphenyl)ethenyl]-4-quinazolinyl]-*N*,*N*-dimethyl-1,3-propanediamine dihydrochloride), a prototype compound, increased the mAb1620 steady state and therefore the active conformation of p53. CP-31398 also shows elongated reporter expression in a p53 activated luciferase reporter constructs transfected into H1299 cells containing mutated p53, reflecting the restoration of the WT p53 function (40). Besides just activating p53, CP-31398 also causes p53 translocation to the mitochondria where it induces a release of cytochrome C which will subsequently initiate apoptosis (41). Further research indicates that the treatment of p53 mutant cells with CP-31398 increases the DNA binding capacity of the DNA binding domain of p53 (42). Other papers published results that indicated that CP-31398 has p53 independent growth-suppressive effects (43). This independent growth suppression is related to elevated ROS generation (44). CP-31398 was also tested for growth inhibition and induction of apoptosis of several HNSCC cell lines (45). While not being tested on HNSCC *in vivo*, CP-31398 has been shown to reduce growth or inhibit progression of different types of cancers such as skin (41), liver (46) and colorectal cancers (47) in animals.

### RITA

RITA “reactivation of p53 and induction of apoptosis”, like nutlin-3, also interrupts the interaction between MDM2 and p53. RITA acts by binding p53 instead of MDM2 like nutlin-3 does (48). To identify the means of interaction between RITA and p53 in the cells, Issaeva et al. used the inherent fluorescent properties of RITA in a fluorescence correlation spectroscopy study. Based on the change in diffusion time (G(τ)) they showed that RITA interacts directly with p53 and does not interact with either MDM2 or GST. It was shown that RITA blocks the interaction of p53 with MDM2 and thus prevents degradation of p53 (48). However, at this time, RITA was not found to actively promote p53 activation through its binding (48). *In vivo* experiments seemed to show that the antitumor effect of RITA is WT p53 dependent (48). On the contrary, in 2011, Roh et al. showed that despite p53 mutational status, RITA induces an antitumor effect in FaDu, an HNSCC cell line containing a mutated form of p53 (45). This was also supported by an earlier paper in which the p53 activity of several different cell lines containing a diverse collection of mutations was tested for their response to RITA (49). This means that RITA can restore function to mutated p53 cells as well as WT p53 cells, and indicates that, in some way, RITA induces a conformational change in p53 as well as inhibits MDM2 from binding and causing degradation of p53 (49). Additionally, while not only causing apoptosis, RITA also seems to induce senescence in the HNSCC cell lines HN30 and HN1 (50). However, when p53 null PCI13 cells were treated with RITA, their clonogenic ability seemed to decrease. This observation points towards a p53-independent effect of RITA that was not described previously (50). Further investigation has also shown that RITA has a p53-independent activation of JNK signalling contributing to apoptosis (51). RITA has been shown to synergize with 3-MA, a PI3K inhibitor, both *in vivo* and *in vitro* in chemotherapy-resistant cells (52).

### PRIMA-1 and APR-246

In 2002, Bykov et al. screened a library of low molecular weight compounds that suppress the proliferation of human cancer cells that contain mutated p53 (53). They discovered the compound 2,2-*bis*(hydroxymethyl)-1-azabicyclo[2,2,2]octan-3-one which was named “p53 reactivation and induction of massive apoptosis” or PRIMA-1. Three years later, they reported a structural analog APR-246 which is the methylated form of PRIMA-1. This modification allows for better diffusion across the cell membrane (54). When either compound enters the cell, the compound gets converted into methylene quinuclidinone (MQ).

MQ binds Cys124 of p53 located in the pocket between loop L2 and sheet S3 in the core domain of p53. This will occur in both WT as well as mutated p53, resulting in a WT conformation of p53 to induce transcription of p53 target genes (55). In addition to Cys124, another important cysteine residue is Cys277 (located in p53 core domain). According to substitution experiments, Cys277 showed to be essential for conserving the thermostabilising function of MQ. This was followed by testing the response of cells with p53 containing the same C277A and C124A substituted residues to APR-246 over time. Apoptotic markers such as Annexin V, p21, Bax and Fas were measured using FACS analysis. This showed that the cells containing either of the two substitutes had markedly less staining of these markers compared to the non-substituted control. These experiments indicate that both residues are essential for the proper reactivation of p53 in cells treated with APR-246 (56). Besides the direct reactivation of p53, APR-246 also induces several p53 independent effects. During the assessment of the antitumor effect of PRIMA-1, a change in the redox balance of the cell was observed. Reactive oxygen species (ROS) levels of the cell were increased while glutathione levels were decreased (55). Due to its sulfhydryl binding properties, MQ binds sulfhydryl groups other than those of p53, such as those in glutathione and antioxidant enzymes such as TrxR1, Prx3 or GPx-1. This in turn causes the accumulation of massive amounts of ROS in the cell synergizing with the reactivation of p53 to induce apoptosis. Furthermore, APR-246 dysregulates the NFE2L2/HMOX axis. This causes the activation of HMOX1 by NFE2L2 resulting in a general protective response that tries to balance the cellular redox. Therefore, knocking down the *NFE2L2* gene causes increased sensitivity to PRIMA-1 (57). To achieve this effect in practice, PI3K or mTOR inhibitors can be used to break down NFE2L2 (57).

In recent years, treatments combined with PRIMA-1 or APR-246 have shown synergistic effects both in *in vitro* and *in vivo* settings. The combination of APR-246 and chemotherapeutics showed a synergistic effect. It is thought that reinstating the WT conformation of p53 may sensitize the cell to chemotherapeutics (54). Additionally, although not shown in head and neck squamous cell carcinoma, PRIMA-1 synergizes with radiotherapy in melanoma (58). Besides combinations with conventional treatments, it has been shown that piperlongumine (59) and PARP-1 (60) inhibitors respectively synergize with and sensitize HNSCC cells to PRIMA-1 and APR-246. Piperlongumine is a compound extracted from the piper longum L. plant which induces cell death both *in vitro* and *in vivo* in pancreatic cancer, breast cancer, leukemia (61). However, this effect is not observed in non-transformed cells. Piperlongumine directly binds to glutathione S transferase Pi 1 causing an increase in intracellular ROS and subsequent apoptotic cancer cell death. In addition to apoptosis, piperlongumine also causes cell cycle arrest, autophagy and inhibits migration and invasion (61). Besides targeting glutathione S transferase Pi 1 directly, docking of piperlongumine to AKT causes a downregulation of AKT phosphorylation both *in vitro* and *in vivo* (62). On the other hand, PARP-1 inhibitor affects the PARP protein primarily implicated in DNA damage repair, and also seem to inhibit TrxR1. This causes a decrease in available glutathione for a breakdown of ROS and therefore results in increased intracellular ROS levels as well (60). Both piperlongumine and PARP-1 inhibitors have functions that influence the redox state of the cells and this is the most likely mechanism in which these compounds synergize with PRIMA-1 and APR-246. Nowadays, several clinical trials have been performed with PRIMA-1 and APR-246, however, none with HNSCC patients.

### Ad-p53

In recent years, viral particles which do not harm normal human cells have been investigated as a form of treatment. These viruses are used to lyse the oncogenic cells and are therefore called oncolytic viruses. Among the viruses being investigated are the herpes simplex virus (63), maraba virus (64) and adenoviral particles. For those types of treatments in this review, we focus on the viral treatments that specifically influence p53 but that is not the only mechanism by which these viral treatments exert antitumoral effects. One treatment termed Ad-p53, also known under the name gendicine, uses an adenoviral particle that can selectively infect both dividing and quiescent cancer cells. The adenoviral vector remains in the cell as an episomal vector which is capable of producing high amounts of the recombinant *TP53* gene transcript. The WT p53, in turn, causes cell death in head and neck cancer cell lines *in vitro* (65). Additionally, treatment with Ad-p53 sensitizes the cells to radiotherapy (66). Finally, in xenograft mice, intratumoral injection of the adenoviral particle causes a significant reduction in tumour size (67).

### ONYX-015

A similar adenovirus called ONYX-015 specifically kills p53 deficient cells. The viral protein E1b55k would normally prevent the activation of p53 allowing the replication of the adenovirus. In ONYX-015, there is an 827bp deletion in the E1B gene region that causes a premature stop codon in E1B55k, this allows p53 sufficient cells to induce apoptosis upon viral infection, preventing full replication of the virus. However, p53 deficient cells, which is often the cases with cancer cells, do not induce apoptosis regardless of the absence of E1B55k. This means that the virus can replicate and causes apoptosis upon accumulation of newly synthesized viral particles. This mechanism allows the virus to specifically infect and kill cancer cells (68, 69). However, later on, it was discovered that ONYX-015 also replicated in WT p53 cells. This seemed to be caused by a loss of p14ARF in those cells. It is thought that this is due to the lack of inhibition of MDM2, the p53 inhibitor. In the absence of p14ARF, MDM2 takes over the role of E1b55k and causes inhibition of p53. This in turn allows the replication of ONYX-015 (70).

## Molecules That Target WTp53 HNSCC

Although many HNSCCs carry a mutated form of p53, 15-25% have WT p53 that is inhibited by overexpressed regulators such as MDM2 and MDMX/4 (3, 71). Different treatments can target this interaction in one of two ways. Either by preventing the inhibitor from binding p53 by occupying the binding site on p53 itself or by removing the inhibitor from p53 by occupying the p53 binding pocket on the inhibitor. Among these treatments are different peptides (72) and small molecules.

### PM2

Although many stapled peptides such as SAH-p53-8 (73) and PM2 (72, 74) are developed and tested on many different cancers, only PM2 has been tested in HNSCC. A stapled peptide differs from standard peptides due to a crosslinking bond between different R- groups from several amino acids forming the peptide. This crosslink allows for a specific helical conformation and improves the stability and resistance of the peptide to protease degradation (75). A stapled peptide will interfere with the protein-protein interaction, which in this case is between p53 and MDM2 and/or MDMX/4.

PM2 is an inhibitor of both MDM2 and MDMX/4 which prevents their binding to p53. PM2 binds 3 residues in the N-terminal hydrophobic cleft of MDM2 thereby preventing its interaction with p53 (75). Since MDM2 is the E3 ubiquitin ligase for p53, its inhibition, therefore, causes accumulation of p53 in the cell (75). Although only tested in a limited amount of head and neck cancers, PM2 synergizes with radiotherapy (76). Additionally, PM2 sensitizes cells to targeted radionuclide therapy (72). Combining PM2 and ^177^Lu-labeled anti CD44v6 antibody (^177^Lu-AbN44v6) increases the antitumor effect when compared to monotherapies. However, this combination requires the expression of CD44v6 which is a cancer stem cell marker in many tumors, and is one of the CD44 isoforms (72).

### Nutlin-3

Nutlins are a group of cis-imidazoline analogs that were discovered in 2004 during a screening for compounds that could inhibit the p53-MDM2 interaction. After testing three nutlins for their IC50 values, Nutlin-3 showed the most potent inhibitory effect. Additionally, it was the arbitrarily assigned enantiomer A that was about 150 times more potent than enantiomer B (IC_50_: 0.09µM vs. 13.6µM). The interaction of the nutlins with MDM2 was determined using crystallography. This shows that the nutlins bind MDM2 in the p53 binding pocket, thereby preventing its binding to p53 itself (77). Using a pan-caspase inhibitor before administration of the active enantiomer caused a reduction in apoptosis, showing that nutlin-3 causes cell death in a caspase-dependent manner involving p53*. In vivo*, Nutlin-3 also showed a high percentage of tumor growth inhibition (77).

In 2010, nutlin-3 was shown to radio sensitize laryngeal carcinoma cells (78). By using several different cell lines with differing p53 mutational status, researchers showed that treating cells with the combination of radiotherapy and nutlin-3 decreased clonogenicity more than with radiotherapy alone. Additionally, cell lines containing WT p53 had increased senescence compared to mutant cells when treated with nutlin-3 combined with radiotherapy compared to radiotherapy alone. This indicates that the radiosensitizing effect of nutlin-3 requires WT p53 (78). In addition to radiosensitizing, Nutlin-3 also enhances the cytotoxicity of chemotherapeutic agents such as cisplatin. Once again, this effect is more pronounced in cells that express WT p53 (45).

Generally, p53 is highly mutated in HNSCC, however, this is not the case for nasopharyngeal cancer where the mutation of p53 is rarer (13%) (79). Therefore, nutlin-3, which requires WT p53 to efficiently exert its function may help improve the outcome for patients with nasopharyngeal cancer. Then, as expected, when testing in nasopharyngeal cancer cell lines, nutlin-3 sensitizes these cells to cisplatin (80, 81).

In recent years, a combination of nutlin-3 and immunotherapy has been explored extensively as well. CD4+ T cells that are reactive against a selected peptide from MDM2 have been developed and showed promising effects against cancer progression. When tested in combination with nutlin-3, the expression of MDM2 in cancer cells was increased which in turn increases the anti-tumour response of the CD4+ T cells (82).

Finally, while not proven in HNSCC, Nutlin-3 has been found to activate anti-apoptotic signalling pathways such as the JNK-pathway which gets activated by p53 and subsequently activates heme oxidase -1 (HO-1). HO-1 may subsequently metabolize heme groups whose metabolites possess anti-apoptotic properties (83). Additionally, p53 may also indirectly phosphorylate MEK and ERK from the RAS/RAF/MEK/ERK pathway which is known to confer anti-apoptotic signals (84). Activation of both pathways involves translocation of activated p53 to mitochondria where it may induce the release of ROS and activation of both JNK as well as MEK and ERK. Finally, Nutlin-3 also interacts with members of the BCL-2 family. However, in which way this affects their function is not well known (85).

### CH1iB

Some drugs target the cellular mechanisms that are specific to HPV-positive HNSCC. The incidence of HPV+-HNSCC increases year over year making this type of treatment more and more relevant. One of these treatments targets the CH1 domain (amino acids 350 to 450) of p300, a co-activator of transcription factor like p53. Besides degrading p53, HPV E6 also associated with p300 preventing the acetylation of p53 (86). Acetylated p53 is more stable and has increased transcriptional activity. Xie et al. (86) found that blocking the interaction between HPV E6 and p300 through an exogenic expression of the CH1 domain could prevent the breakdown of p53 due to competitive inhibition of HPV E6 by the exogenic CH1 (86).

Xie et al. transfected HPV+ cells (UMSCC47 and UPCI : SCC090) with an exogenous CH1 polypeptide (86). The evaluation of p53 in these cell lines showed that the transfected cells had more acetylated p53 than the non-transfect cells. The transformation of cells with exogenous CH1 showed a significant decrease in clonogenic survival compared to the control. Ectopic expression of the CH1 domain of p300 in HPV-positive HNSCC also sensitized the cells to cisplatin (86). In HPV- cells, the same results were obtained as p300 also binds MDM2. Therefore, exogenous CH1 also blocks the interaction between p300 and MDM2 which subsequently increases the p53 activity (86). To implement this principle in the clinic, a helical mimic named CH1iB inhibiting the p300-HPV E6 enzyme interaction has been developed. However, it is important to note that only inhibition of binding site B of CH1 with the mimic CH1iB showed to be efficacious. CH1iB was reported to reduce the interaction between p300 and HPV E6 thus allowing p300 to acetylate p53 which resulted in an increase in p53 transcriptional activation (86, 87).

## Molecules That Specifically Target HPV^+^ HNSCC

### Ad-E6/E7-AS

Like Ad-p53 & ONYX-015 earlier in this paper, other adenovirus particles are used in combatting HNSCC. An adenoviral particle, first tested in cervical cancer cells, transfers the HPV16 E6/E7 antisense RNA (Ad-E6/E7-AS) (88). When Ad-E6/E7-AS infects the HPV+ cell, the antisense RNA will hybridize with the RNA for HPV proteins E6 and E7. The new dsRNA will be degraded by the cell preventing translation of the E6 and E7 transcript. Therefore, the p53 protein which is normally degraded is then expressed at a normal level due to the absence of the proteasomal breakdown originally caused by the HPV-derived E6 protein. When treated with Ad-E6/E7-AS, the growth of the head and neck cancer cell line SCC47 was stopped. Additionally, it was also tested in combination with cisplatin which showed a synergistic effect with Ad-E6/E7-AS (89).

### Bortezomib

In 1995, Muogenics developed a molecule called Bortezomib. A compound that targets the proteasomal 26S complex and thereby also preventing the breakdown of p53 by either MDM2 or HPV E6. It increases apoptosis in HPV+ cell lines but does not affect sensitivity to chemo- or radiotherapy (90). In addition to the inhibition of p53 breakdown, bortezomib also inhibits the downregulation of IkB. The release of the p65-p50 complex is subsequently also inhibited and will prevent activation of the NF-kB target genes resulting in decreased proliferation and cell survival (91). In addition, phosphorylation of AKT is also decreased due to increased activity of the phosphatase PP2A which results in reduced signalling to mTOR contributing to bortezomib-induced apoptosis. Bortezomib has subsequently been shown to decrease proliferation both *in vitro* as well as *in vivo* (92).

### Triptolide and Minnelide

Triptolide is a molecule that was originally isolated from the Chinese herb *Tripterygium wilfordii*. Ever since it has been extensively studied in several types of diseases and cancers (93), (94). Its more water-soluble derivative Minnelide, prevents E6 transcription thereby also preventing the breakdown of p53 and thus resulting in massive apoptosis (95). Minnelide has also been shown to induce apoptosis and helps overcome cisplatin resistance in PDXs *in vivo* (96). Additionally, Minnelide inhibits HSP70 and reduces the secretion of IFN-y resulting in decreased PD-L1 expression.

## Targeting p53 Through Macrophage Polarization

Many studies have shown the involvement of immune cells in the progression of HNSCC, and tumor-associated macrophages (TAMs) are an important element of the TME of HNSCC promoting multiple aspects of cancer development. In HNSCC patients, it is now demonstrated that high density of TAMs correlates with poor prognosis (97). Furthermore, 80% of stained macrophages in the TME are macrophages of M2 phenotype, namely TAMs (98). It is increasingly clear that the polarization of macrophages into TAMs and their infiltration in the TME is a poor prognostic factor, making these TAMs a promising target for HNC patients. A first model of chronic liver damage has initially demonstrated that proliferating stellate cells deficient for p53 were able to stimulate polarization of macrophages into a tumor-promoting M2-state and enhance the proliferation of premalignant cells (99) Since then, many studies focused on the clinical and prognostic implication of p53 on macrophage infiltration in some cancers and revealed that patients with mutant TP53 had significantly higher macrophage infiltration than those with wild-type TP53 and this was correlated with poorer survival (100). Moreover, a recent study on bladder carcinoma highlighted the association between M2 macrophage polarization and p53 dysfunction (101). Interestingly, Fang et al. recently tested a pharmacological p53 activator in combination with PD-1 blockade and investigated the role of p53 on immune modulation. Their results were very encouraging as they demonstrated that p53 activation promotes antitumor immunity in the TME, with a decrease in M2 macrophages, both in TP53 wild-type and TP53 mutated mice tumors (102).

## Targeting p53 in HNSCC in the Clinical Setting

Although many treatments have been tested in preclinical settings, only some have reached clinical trials in treating HNSCC patients. Adenoviral particles are most commonly used in clinical trials. The only small compound that is currently tested in clinical studies involving HNSCC is COTI-2, which is used in combination with cisplatin in a phase II trial. However, no results have been posted yet (NCT02433626).

The most researched aforementioned treatment is the Ad-p53. While it is not yet used in western clinics, Ad-p53 was approved in October of 2003 by the Chinese food and drug administration under the name gendicine (103). Between the years 2000 and 2010, multiple clinical trials using combinations of gendicine and radiotherapy or chemotherapy have been performed. Both the total response rate as well as the overall 1-, 2-, 3- and 5-year survival rates were significantly improved when gencidine was added to the treatment compared to the conventional treatment alone (104). One clinical trial shows that the complete response of the combination of gencidine and radiotherapy was increased 2.1 fold compared to radiotherapy alone (62.5% versus 29.6%). Additionally, the 3-year overall survival was 14.4% higher in the combination group. Currently, for HNSCC, 4 clinical trials currently evaluate the efficacy of Ad-p53 either alone or in combination with other treatments such as immune checkpoint inhibitors or the standard of care, concomitant chemoradiotherapy (NCT02842125, NCT00003257, NCT00017173, NCT03544723).

Similarly, ONYX-015 has also been used in a clinical setting in HNSCC patients. A 2016 Phase II clinical trial showed ONYX-015 to be safe for use. In the same study, it is suggested that the treatment also shows modest antitumor activity (105). The last Phase III trial using ONYX-015 in combination with cisplatin and fluorouracil was withdrawn in 2013 without posting any results (NCT00006106).

## Conclusion

This review discusses the current status of pharmacological manipulation of p53 for the treatment of HNSCC. Due to the need for new treatment combinations in HNSCC, many studies have looked at different p53 reactivators for an answer. The three described strategies to reactivate p53 in HNSCC are proposed regarding to the mutational status of p53 and the infection by HPV, which lead to a new classification of HNSCC encompassing WT p53, mutated p53 and HPV positive cancer cells. Such subtypes may be associated with effective and specific treatment strategies that must be optimized before clinical application. While some of these drugs (COTI-2 and adenoviral particles) have been tested in clinical settings, more research is needed to find more specific, safe and effective p53 reactivators to improve the treatment of HNSCC.

## Author Contributions

TB and MK collected the data and drafted the manuscript. DG, FJ, and GD revised the manuscript. TD, SS, and GG provided additional revisions. All authors contributed to the article and approved the submitted version.

## Funding

This work has been supported by “Les Amis de l’Institut Jules Bordet”.

## Conflict of Interest

The authors declare that the research was conducted in the absence of any commercial or financial relationships that could be construed as a potential conflict of interest.

## Publisher’s Note

All claims expressed in this article are solely those of the authors and do not necessarily represent those of their affiliated organizations, or those of the publisher, the editors and the reviewers. Any product that may be evaluated in this article, or claim that may be made by its manufacturer, is not guaranteed or endorsed by the publisher.
